# Host-Derived Microvesicles Carrying Bacterial Pore-Forming Toxins Deliver Signals to Macrophages: A Novel Mechanism of Shaping Immune Responses

**DOI:** 10.3389/fimmu.2018.01688

**Published:** 2018-07-27

**Authors:** René Köffel, Heidi Wolfmeier, Yu Larpin, Hervé Besançon, Roman Schoenauer, Viktoria S. Babiychuk, Patrick Drücker, Thomas Pabst, Timothy J. Mitchell, Eduard B. Babiychuk, Annette Draeger

**Affiliations:** ^1^Institute of Anatomy, University of Bern, Bern, Switzerland; ^2^Department of Medical Oncology, University Hospital Bern, Bern, Switzerland; ^3^College of Medical and Dental Sciences, Institute of Microbiology and Infection, University of Birmingham, Edgbaston, Birmingham, United Kingdom

**Keywords:** macrophage polarization, microvesicles, liposomes, bacterial pore-forming toxins, host-defense

## Abstract

Bacterial infectious diseases are a leading cause of death. Pore-forming toxins (PFTs) are important virulence factors of Gram-positive pathogens, which disrupt the plasma membrane of host cells and can lead to cell death. Yet, host defense and cell membrane repair mechanisms have been identified: i.e., PFTs can be eliminated from membranes as microvesicles, thus limiting the extent of cell damage. Released into an inflammatory environment, these host-derived PFTs-carrying microvesicles encounter innate immune cells as first-line defenders. This study investigated the impact of microvesicle- or liposome-sequestered PFTs on human macrophage polarization *in vitro*. We show that microvesicle-sequestered PFTs are phagocytosed by macrophages and induce their polarization into a novel CD14^+^MHCII^low^CD86^low^ phenotype. Macrophages polarized in this way exhibit an enhanced response to Gram-positive bacterial ligands and a blunted response to Gram-negative ligands. Liposomes, which were recently shown to sequester PFTs and so protect mice from lethal bacterial infections, show the same effect on macrophage polarization in analogy to host-derived microvesicles. This novel type of polarized macrophage exhibits an enhanced response to Gram-positive bacterial ligands. The specific recognition of their cargo might be of advantage in the efficiency of targeted bacterial clearance.

## Introduction

During infection, membrane damaging toxins are released by numerous bacterial pathogens and contribute significantly to their virulence (1). An important family of membrane perforating toxins are the cholesterol-dependent cytolysins (CDCs) consisting of more than 20 members, which are secreted by Gram-positive bacteria. Notable representatives are pneumolysin (PLY, from *Streptococcus pneumoniae*), and streptolysin O (SLO, from *Streptococcus pyogenes*), which insert into host cell membranes and form large oligomeric pores that allow the leakage of cytoplasmatic proteins (2, 3). CDCs are secreted as water soluble molecules that become associated with the host cell membrane either *via* a specific receptor and/or cholesterol-rich microdomains (4). Subsequently, the large multimers undergo structural changes and form large aqueous pores resulting in Ca^2+^ influx and cytoplasmic efflux (3).

In order to prevent cell death, a concerted action of Ca^2+^-sensing protein, either directly repair the plasmalemma or activate the membrane repair machinery (5). Recent work from our laboratory showed that—in response to the rise in cytoplasmic Ca^2+^—the annexins, members of a phospholipid-binding protein family translocate to the plasmalemma, quarantine the toxin pore within a membrane fold, which is then shed into the extracellular space (6). By this route, PLY-pores are actively removed from epithelial cell membranes (7). FACS analysis and cryo-electronmicroscopy of PLY-microvesicles confirm an association of PLY-pores and annexin family members and demonstrated that 90% of the vesicles were smaller than 500 nm with a median size of 160 nm (7).

Myeloid immune cells (monocytes, macrophages, dendritic cells, and granulocytes) are first line defenders in infections and coordinate the host’s immune responses to pathogenic threats. Inflammation in bacterial infection is often triggered by pore-forming toxin-induced epithelial damage and leads to the release of cytokines that activate and recruit immune cells (8). The innate immune system recognizes pathogenic microorganisms by pathogen-associated molecular patterns (PAMPs) *via* pattern recognition receptors (PRRs). PAMPs [e.g., lipopolysaccharide (LPS); peptidoglycan (PGN); bacterial lipoproteins; unmethylated repeats of CpG nucleic acids] represent molecular structures or molecules that are shared by most pathogenic microorganisms. Detection of PAMPs by PRRs such as toll-like receptors (TLRs), retinoic acid-inducible gene-I-like receptors (RLRs), and nucleotide-binding oligomerization domain (NOD)-like receptors (NLRs), triggers an immune response. For example, cell wall component LPS from Gram-negative bacteria is recognized by TLR4. The PRR for PGN, an important structural component of bacterial cell walls from mostly Gram-positive bacteria, is still a matter of debate. In mammals, there are two NLRs for recognition of PGN. NOD2 detects (Lys)-type PGN and muramyl dipeptide (MDP) as the minimal recognition structure, whereas NOD1 preferentially senses the diaminopimelate-containing GlcNAc-MurNAc tripeptide muropeptide found mostly in Gram-negative PGN (9–11). While the role of NOD2 as a receptor for Gram-positive PGN is well documented, reports as to the role of TLR2 as a PGN receptor are contradictory. In some studies, TLR2 is described as a receptor for PGN (12–16). Other reports show that both MDP and highly purified PGN from several bacteria were not detected by TLR2 (17–19). Thus, there is a tendency to believe that TLR2 is not stimulated by PGN. The response of innate immune cells after PRRs ligation includes enhanced phagocytosis and cytokine production in monocytes/macrophages and neutrophils. It is not surprising that the responses of local immune cells (e.g., macrophages) and immediately recruited neutrophils and monocytes, are constitutively shaped by signals present at the site of infection (20). For instance, PAMPs and danger-associated molecular patterns (DAMPs); which are released by damaged epithelial cells, have been shown to differentially polarize innate immune cells and thus shape the type of immune response (21). Polarization of macrophages leads to a modulation of their functional properties, ranging from pro-inflammatory (M1 phenotype) to anti-inflammatory (M2 phenotype) (22). These polarizations are not only driven by cytokines [i.e., interferon-γ (IFN-γ) and interleukin-4 (IL-4)] but also by immune complexes, and/or bacterial constituents (i.e., LPS) (23). M1-macrophages express high levels of pro-inflammatory cytokines [i.e., interleukin-1β, IL-1β, interleukin-6, IL-6, and tumor necrosis factor α (TNFα)] and participate in the induction of a Th1 response to prevent pathogen persistence. In contrast, M2-macrophages express different sets of receptors (i.e., CD206 and CD163) and produce primarily anti-inflammatory cytokines (i.e., interleukin-10, IL-10) (24). Thus, in broad sense, M1-macrophages act microbicidial and pro-inflammatory, whereas M2-macrophages are immunosuppressive. Recently, inflammation-driven polarization was also shown for neutrophils as they can acquire a monocytic phenotype and antigen-presenting cell functions (25, 26).

Plasma membrane repair results in the active shedding of copious amounts of plasma membrane particles containing inactivated toxins (6, 7). Despite the detailed elucidation of membrane repair mechanisms, it is not known whether the released microvesicles or the liposomal decoys that contain PFTs are able to activate immune cells (6, 7). In this study, we have analyzed the influence of inactivated PFTs, i.e., PLY, on the host’s innate immune system: taken up by host macrophages, microvesicles carrying neutralized bacterial toxins display immunomodulatory effects, which lead to a hitherto unknown pattern of macrophage polarization. Their change in phenotype enables host macrophages to mount a specific response to Gram-positive inflammatory stimuli while Gram-negative stimuli are dampened. This novel macrophage phenotype might be primed to play a role in the rapid and efficient clearance of distinct classes of bacterial pathogens.

## Materials and Methods

### Reagents

Macrophage colony-stimulating factor (M-CSF), interleukin-4 (IL-4), and IFN-γ were purchased from BioLegend (London, UK). LPS, PGN, and SLO were purchased from Sigma-Aldrich. Pam2CSK4 was purchased from InvivoGen (Toulouse, France). PLY was prepared as described (7) and bacterial endotoxins from protein stocks were removed by endotoxin removal resin (ThermoFisher Scientific, Reinach, Switzerland). Non-hemolytic ΔPLY protein mutant (ΔA146R147; amino acid deletions A146, R147) is described elsewhere (27). Liposomes (Cal02) used in these studies were a kind gift of Combioxin SA (Geneva, Switzerland).

### Ethics Statement

Human buffy coats from healthy volunteers were purchased from the RedCross Switzerland (Bern, Switzerland). All subjects gave written informed consent in accordance with the Declaration of Helsinki. The buffy coats are pre-existing materials that were purchased with the ethics committee approval of the RedCross Switzerland (project P_103 to René Köffel). All obtained samples were anonymized, thus no subject-identifying information was associated with the buffy coats.

### Cells

Primary human monocytes from buffy coats were isolated as previously described (25) and differentiated to macrophages in RPMI supplemented with 10% heat-inactivated fetal bovine serum (FBS) (Gibco, Life Technologies, Paisley, UK) and 1% penicillin–streptomycin (Gibco, Life Technologies, Paisley, UK) with M-CSF (75 ng/ml) for 7 days. Human embryonic kidney cells (HEK 293; ATCC CRL-1573; Manassas, VA, USA) were maintained in DMEM supplemented with 10% heat-inactivated FBS and 1% penicillin–streptomycin. Cell cultures were grown in 5% CO_2_ at 37°C.

### Bacterial Culture

*Streptococcus pneumoniae D39* (laboratory strain) was cultured in BHI (Brain Heart Infusion Broth, Sigma-Aldrich, Buchs, Switzerland) at 37°C. For the generation of bacterial supernatants, bacteria grown to their stationary phase (OD_600_ = 1.0) were pelleted (5,000 × *g*) for 15 min. The supernatants were filtered through a syringe filter with a pore size of 0.2 µm (VWR, Dietikon, Switzerland) and incubated with liposomes (Cal02) to remove pore-forming toxin activity before re-stimulation experiments.

### Isolation of PLY-Induced Microvesicles

HEK 293 cells (10^7^) were gently washed with PBS. Microvesicle shedding was induced by treatment with PLY (2 µg/ml) in Ca-Tyrode’s buffer (140 mM NaCl, 5 mM KCl, 1 mM MgCl_2_, 10 mM glucose, 10 mM HEPES, pH7.4, 2.5 mM CaCl_2_). The cells were incubated for 45 min at room temperature. Shed microvesicles were isolated according to a protocol for extracellular vesicles (28). Briefly, conditioned medium was centrifuged at 300 × *g* for 10 min at 4°C to pellet cells. Supernatant was centrifuged at 2,000 × *g* for 20 min at 4°C (2 k pellet), transferred to new tubes, and centrifuged in a 45 Ti rotor for 40 min at 10,000 × *g* (10 k pellet), and finally in a 90 Ti rotor (Beckman Coulter, Nyon, Switzerland) for 90 min at 100,000 × *g* (100 k pellet). The pellets were re-suspended in Ca-Tyrode’s buffer for stimulation of macrophages.

### Polarization of Macrophages

After 7 days, media from macrophage cultures was exchanged to RPMI supplemented with 2% heat-inactivated FBS and 1% penicillin–streptomycin. Polarization of the macrophages was induced as described (29). Briefly, polarization was induced by 100 ng/ml LPS plus 25 ng/ml IFN-γ (for M1 state), 20 ng/ml IL-4 (for M2 state), PLY-microvesicles, PLY-liposomes, or liposomes only for 48 h.

### Flow Cytometry

Flow cytometry analysis was performed as described (25). The following murine monoclonal antibodies (mAbs) were used for FACS: unconjugated mAbs for TLR2 and TLR4; FITC-conjugated mAbs specific for CD206, CD83; phycoerythrin (PE)-conjugated mAbs specific for CD86, allophycocyanin-conjugated mAb specific for CD14; PerCP-Cy5.5-conjugated mAbs specific for CD163; PE-Cy7-conjugated mAb specific for HLA-DR. All antibodies and istotype control mAbs were purchased from BioLegend (London, UK). FACS analyses were carried out on LSRII cytometers (BD Instruments, San Jose, CA, USA). Data were analyzed with FlowJo software (LLC, Ashland, OR, USA).

### Western Blot

Proteins from equal numbers of cells were separated by SDS-Page and blotted onto polyvinylene difluoride membrane (PVDF) (Millipore, Billerica, MA, USA). For preparation of whole cell extracts, cells resuspended in sample buffer (0.5 M Tris–HCl pH 6.8; 40% glycerol; 4% SDS; 5 µl/ml bromphenolblue; 5% beta-mercaptoethanol) and lysed by heating at 95°C for 10 min. Proteins resolved by SDS-PAGE were transferred to PVDF membranes, which were probed with monoclonal anti-PLY (Santa Cruz) followed by horseradish peroxide-conjugated goat anti-mouse IgG (H + L) antibodies (GE healthcare, Little Chalfont, UK). Protein detection was performed with the chemiluminescent substrate SuperSignal Quantum (Advansta, Menlo Park, USA) on a luminescent image analyzer (Witec AG, Switzerland).

### Real-Time RT-PCR

Total RNA was isolated using the RNeasy Micro Kit (Qiagen, Hilden, Germany). For real-time RT-PCR analysis, the SYBR Green detection system was used (Quanta Biosciences, Beverly, MA, USA). All primers were designed using the Primer3 design tool. The following primer pairs were used for RT-PCR: TNFα 5′-AGCCTCTTCTCCTTCCTGATCGTG-3′ (forward)/5′-GGCTGATTAGAGAGAGGTCCCTGG-3′ (reverse); CCL5 5′-TACACCAGTGGCAAGTGCTC-3′ (forward)/5′-TGTACTCCCGAACCCATTTC-3′ (reverse); SOCS1 5′-TGTTGTAGCAGCTTAACTGTATC-3′ (forward)/5′-AGAGGTAGGAGGTGCGAGT-3′ (reverse); SOCS3 5′-CACTCTTCAGCATCTCTGTCGGAAG-3′ (forward)/5′-CATAGGAGTCCAGGTGGCCGTTGAC-3′ (reverse); STAT1 5′-TGGGTTTGACAAGGTTCTT-3′ (forward)/5′-TATGCAGTGCCACGGAAAG-3′ (reverse); GAPDH 5′-GAAATCCCATCACCATCTTCCAGG-3′ (forward)/5′-CGCGGCCATCACGCCACAGTTTCC-3′ (reverse); NOD1 5′-CAGAGTCTCACCCCCACATT-3′ (forward)/5′-CGGCCGAGAAGTAGTCATTC-3′ (reverse); NOD2 5′-ATCTTCACACCGTCCCAGAG-3′ (forward)/5′-GCCAATGGGACTGGTAATTC-3′ (reverse). Expression profiling was performed in a 96-well format on the StepOne™ Real-Time PCR System (Applied Biosystems, Foster City, CA, USA). Data were analyzed using StepOne software for relative quantification. The expression of target genes was normalized to GAPDH.

### Immunohistochemistry

5 × 10^4^ monocytes were differentiated to macrophages in chamberslides (Lab-Tek, Germany) for 7 days with M-CSF (75 ng/ml), fixed with 2% paraformaldehyde and permeabilized with ice-cold acetone. The cells were labeled with a polyclonal antibody against NFkB (Santa Cruz Biotechnology, USA) and a polyclonal, Cy3-tagged secondary antibody (Jackson Immunoresearch, Suffok, UK). Nuclei were stained with Hoechst 33248, and slides were mounted using mounting medium (Agilent, Santa Clara, CA, USA). Cells were analyzed using a confocal microscope Zeiss LSM880 and imaged using Zen software (Zeiss, Germany).

### Cytokine Measurement

Screening for cytokines secreted from macrophages was performed by hybridizing conditioned medium with antibody-coated membranes (human cytokine antibody array-membrane, Abcam, Cambridge, MA, USA) according to the manufacturer’s instructions. Briefly, macrophages were stimulated for 48 h and the culture supernatants were pooled and then hybridized to the array membrane. A biotin-conjugated second antibody was used and cytokines were detected by HRP-conjugated streptavidin. Signals were quantified by a luminescent image analyzer (Witec AG, Switzerland). Additional quantifications were performed using the Bio-Plex Pro™ assay according to manufacturer’s protocol. Measurement was performed on a Bio-Plex100 with Bio-Plex Manager 6.1 software (Bio-Rad, Hercules, CA, USA).

### Phagocytosis Assay

A phagocytosis assay was performed using *E. coli-*expressing EGFP-PLY as described (25). In brief, macrophages (5 × 10^5^ each) were incubated with EGFP-*E. coli* for 30 min at 37°C and on ice and phagocytic capacity was determined by FACS after quenching of surface bound fluorescent signal with 0.02% Trypan-blue. In addition, microscopic examination confirmed intracellular EGFP-*E. coli* signals.

### Electron Microscopy

Cells were fixed with 2% paraformaldehyde, 2.5% glutaraldehyde in 0.1 M sodium cacodylate buffer, pH 7.2, washed in the same buffer, and post-fixed in 1% potassium ferrocyanide reduced osmium textroxide as described (30). The samples were then dehydrated through a series of ethanol. Epon-embedded ultra-thin sections were examined with a Tecnai spirit (FEI, Eindhoven, Netherlands).

### Statistics

Numerical data are expressed as mean values together with the SE. The statistical analyses were performed using GraphPad Prism7 software (GraphPad Software, La Jolla, CA, USA). Significant differences are marked with asterisks.

## Results

### Stimulation of Human Macrophages With Host-Derived PLY-Bearing Microvesicles Induces Phenotypic Changes

**Figure 1:**
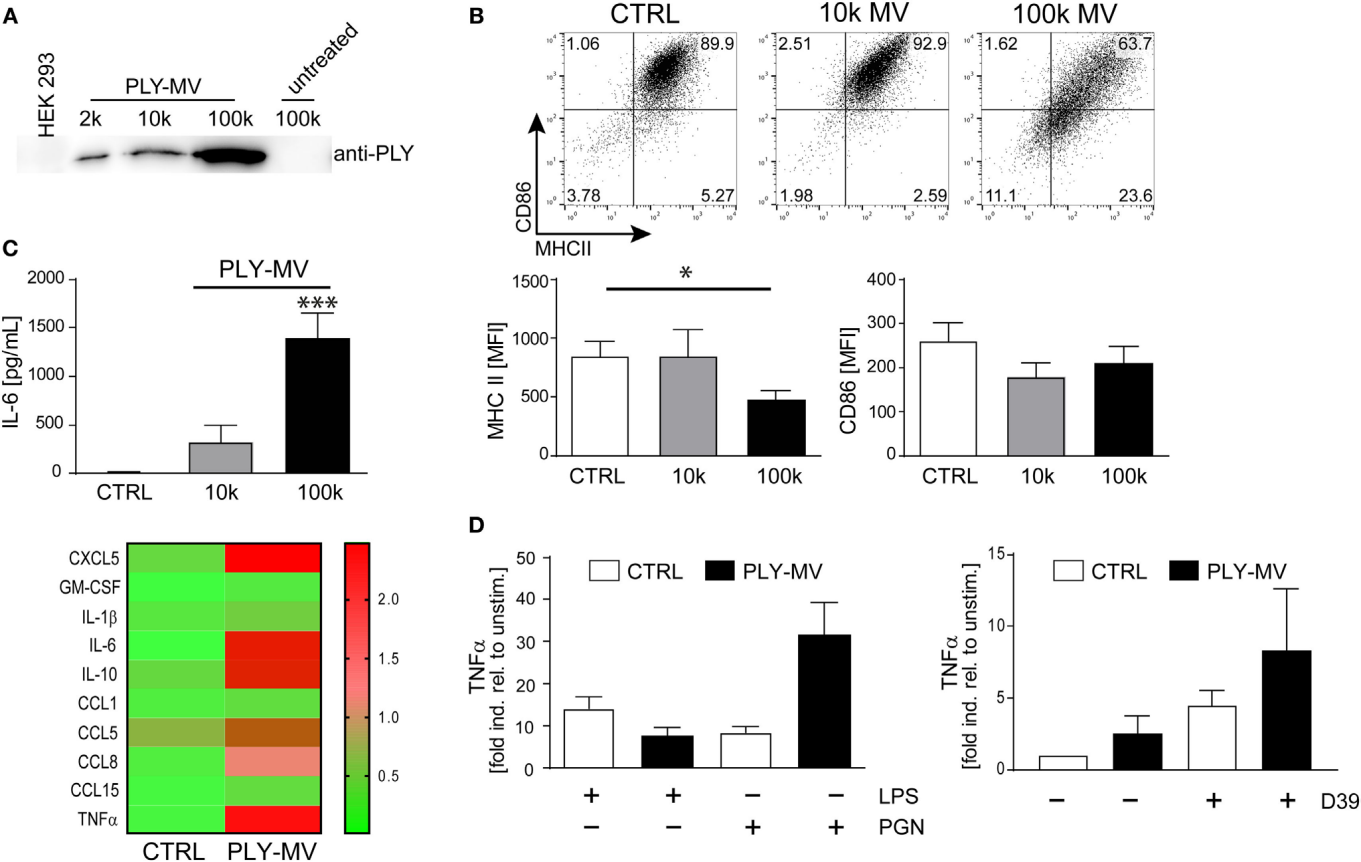
Pneumolysin (PLY)-induced microvesicles generate phenotypic changes in human macrophages *in vitro*. PLY-pores shed on microvesicles (PLY-MV) were generated by HEK-293 cells by stimulation with 2 µg/ml purified PLY for 30 min. This protocol, using a sub-lytic PLY concentration, ensures efficient membrane repair induction in HEK-293 cells without causing substantial cell death (7). **(A)** Isolation of PLY-MV. PLY-MV were isolated by differential centrifugation from cell culture supernatants and fractions (2 k, 2,000 × *g* pellet; 10 k, 10,000 × *g* pellet; 100 k, 100,000 × *g* pellet) were analyzed by Western blotting with a monoclonal antibody against PLY. One representative blot of three independent experiments is shown. **(B)** Stimulation of macrophages with PLY-MV. Day 7 M-CSF induced human macrophages were stimulated with PLY-microvesicles. FACS analysis after 48 h stimulation showed a marked downregulation of MHCII and CD86 in CD14^+^ gated macrophages treated with PLY-MV (100 k MV) as compared to untreated controls (CTRL) or 10 k MV-treated cells. A representative FACS blot is shown. Mean fluorescence intensity (MFI) of MHCII and CD86 of CD14^+^ gated cells were subjected to statistical analysis (100 k MV; mean ± SD; *n* = 5; *p < 0.05; unpaired *t*-test; two-tailed). **(C)** Cytokine profile of PLY-MV induced macrophages. Macrophages were stimulated as in **(A)** and IL-6 production was determined after 48 h from culture supernatants using Bio-Plex (BioRad, Mean ± SD; *n* = 5; ***p < 0.001; unpaired *t*-test; two-tailed) or for global cytokine analysis Abcam cytokine arrays with pooled supernatants from three independent experiments were performed (heat-map; lower panel). Densitometric analysis were accomplished using the ImageJ protein analyser macro [written by G. Carpentier, 2010; http://rsb.info.nih.gov/ij/macros/toolsets/Protein%20Array%20Analyzer.txt]. Only cytokines with at least threefold difference between CTRL and 100 k PLY-MV were evaluated. **(D)** Re-stimulation of PLY-MV induced macrophages. Re-stimulation was performed with 100 ng/ml LPS, 1 µg/ml peptidoglycan, or supernatant from *Streptococcus pneumoniae* strain *D39* (*D39*) for 6 h. RNA was isolated at time points 0 and 6 h. RT-PCR analysis was performed using primers for TNFα and SYBR green RT-PCR kit. Glyceraldehyde 3-phosphate dehydrogenase (GAPDH) served as a reference gene (mean ± SD *n* = 5).

Ectosomes and membrane vesicles are implicated in short and long distance intercellular communication and are attributed with immunomodulatory effects by acting primarily on immune cells (31–33). Recent results showed that microvesicles (below 500 nm of size), with inactivated toxin pores are released during membrane repair aiding cellular survival (7). We isolated PLY-microvesicles according to a recently established protocol by high-speed centrifugation and cryo-electronmicroscopy confirmed an association of PLY-pores with microvesicles (7, 28). Western blot analysis showed the presence of PLY-pores in the 10 k fraction. However, enrichment of PLY-pores was particularly noticeable in the 100 k fraction (Figure 1A). Next, we stimulated macrophages either with the 10 k or the 100 k fraction and analyzed their phenotype after 48 h. Macrophages stimulated with the 10 k microvesicle fraction showed a slight, albeit not significantly higher expression of MHCII. In contrast, the 100 k microvesicles fraction induced a CD14^+^MHCII^low^ phenotype (Figure 1B). In addition, a non-significant downregulation of the mean fluorescence intensity (MFI) of CD86, was observed. Moreover, the stimulation with PLY-microvesicles (100 k) induced a strong secretion of IL-6 and a moderate one after stimulation with the 10 k fraction (Figure 1C). PLY-microvesicle induced macrophages also produced high amounts of IL-1β, TNFα, CCL5, CCL8, and CCL1, which resembles a pro-inflammatory macrophage signature (Figure 1C, lower panel). In contrast to the pro-inflammatory signature, these macrophages showed a downregulation of MHCII and co-stimulatory receptor CD86, which is attributed to M2-polarization (Figure S1 in Supplementary Material). However, a hallmark of M2-polarization, such as upregulation of CD206 and CD163, was not observed in these cells. Next, we re-stimulated PLY-microvesicle-induced CD14^+^MHCII^low^ macrophages with PGN (Gram-positive ligand) or LPS (Gram-negative ligand). These analyses reveal a reduced immediate TNFα response after 6 h LPS re-stimulation in PLY-microvesicle induced macrophages as compared to controls. Moreover, the re-stimulation with PGN induced higher TNFα levels in CD14^+^MHII^low^ macrophages, which even exceeded the TNFα induction in PGN stimulated control cells (Figure 1D). Importantly, PLY-microvesicle induced macrophages also showed an enhanced immediate TNFα response to re-stimulation with supernatants from *S. pneumoniae* (Figure 1D). These bacterial supernatants contain a whole spectrum of Gram-positive PRR/TLR ligands.

Taken together, our results demonstrate that PLY-pores bound to microvesicles induce macrophage polarization to a specific CD14^+^MHCII^low^ phenotype. The cytokine response of these macrophages (i.e., increased TNFα) is specifically directed against Gram-positive bacterial ligands.

### Uptake of Sequestered PLY by Human Macrophages

**Figure 2:**
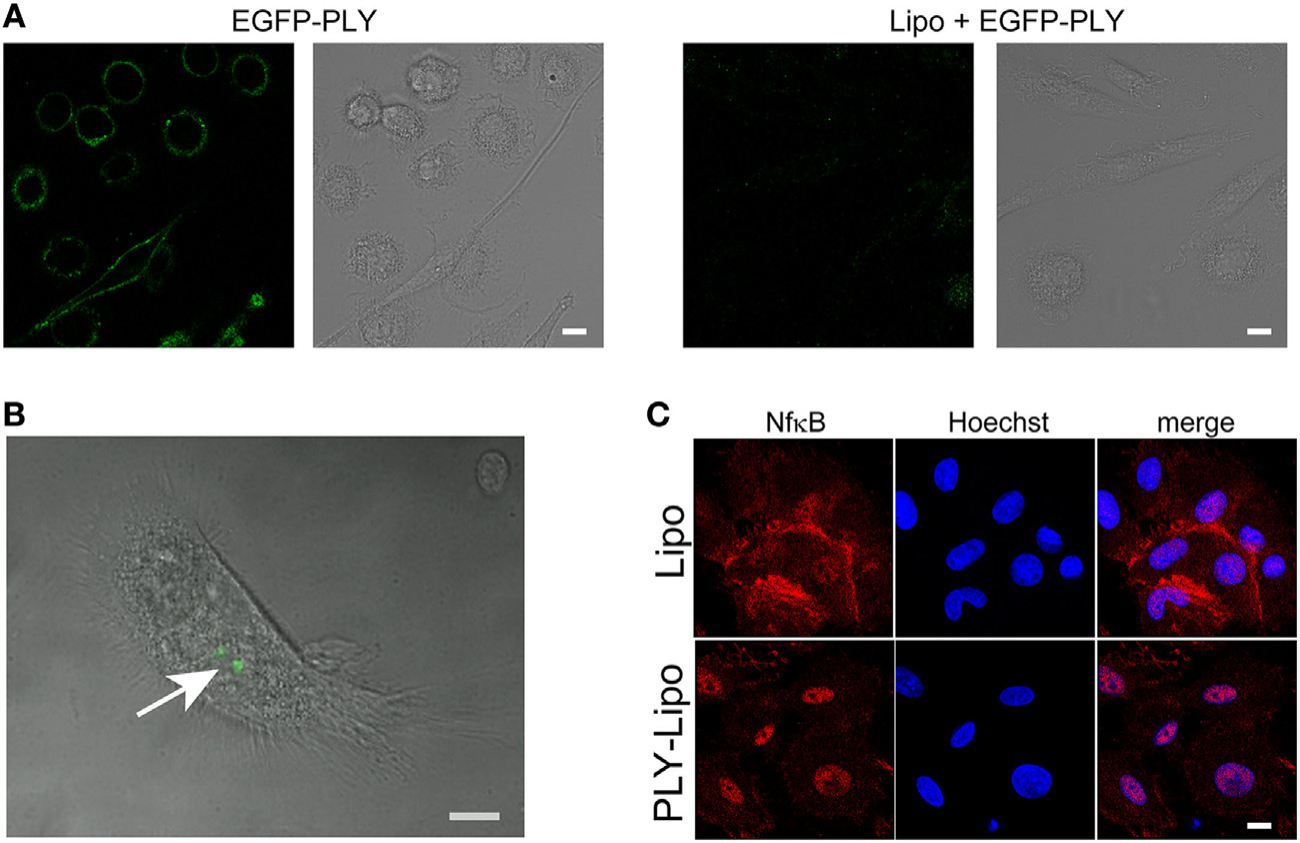
Human macrophages sense pneumolysin (PLY)-bearing liposomes. **(A)** PLY does not bind to macrophages in the presence of cholesterol:sphingomyelin liposomes. Day 7 M-CSF differentiated macrophages, either pre-incubated with liposomes (Lipo) or left untreated, were stimulated with 4 µg/ml EGFP-PLY and serial confocal imaging was performed. Images from *T* = 2 min post PLY additions are shown. The toxin binds exclusively to untreated macrophages. **(B)** Uptake of sequestered PLY on liposomes by human macrophages *in vitro* (arrow). Human primary macrophages were stimulated with EGFP-PLY-liposomes and analyzed at *T* = 4 h by confocal microscopy for intracellular EGFP-PLY (bar 10 µm). **(C)** Stimulation of macrophages with PLY-Lipo induces NfkB translocation from the cytoplasm into the nucleus. Day 7 M-CSF differentiated macrophages were stimulated with PLY-Lipo for 4 h. Afterward, cells were fixed, permeabilized, and stained with a monoclonal antibody against NfkB (red) and Hoechst (chromatin, blue).

Liposomes can prevent host cell damage by sequestering PFTs and protect mice from lethal pneumococcal infections (34). In analogy to macrophage sensitivity to host-derived PLY-microvesicles (Figure 1), we investigated the polarizing effects of toxin-sequestering liposomes. Cholesterol-sphingomyelin liposomes were recently been shown to be able to sequester pore-forming toxins (PFTs), i.e., PLY or SLO, and thus protect THP-1 cells from lysis in *in vitro* experiments (34). Initially, we monitored the efficacy of these liposomes to protect macrophages from cell death by EGFP-tagged PLY. Stimulation of macrophages with EGFP-PLY without liposomes showed rapid binding of PLY to the plasma membrane. In contrast, addition of liposomes before stimulation with EGFP-PLY completely abolished toxin binding (Figure 2A). Liposome-protected macrophages displayed intracellular accumulations of EGFP-PLY at later time points, which suggested phagocytosis of toxin-bearing liposomes. In order to monitor toxin uptake, liposomes preincubated with EGFP-PLY were used to stimulate macrophages. FACS and microscopic analysis showed that PLY-liposomes are phagocytosed by macrophages within 1–4 h after co-incubation (Figure 2B; Figure S2A in Supplementary Material). Ultrastructural analysis of macrophages stimulated with PLY-liposomes showed an increased number of lysosomes in these cells as compared to liposome-alone stimulated macrophages, thus suggesting an activated macrophage phenotype (Figure S2B in Supplementary Material) (35). NfkB represents a key factor in the activation of macrophages that can trigger either M1 or M2 macrophage polarization (36, 37). NfkB translocated to the nucleus only after stimulation of macrophages with PLY-liposomes for 4 h whereas liposomes without toxin (=empty liposomes) did not elicit NfkB translocation (Figure 2C). These data suggest that PLY-pores bound to liposomes can be phagocytosed and are sensed by host macrophages. Importantly, since such inactivated PLY-pores lack cell damaging capacity, they are able to transmit signals to macrophages without danger.

### Stimulation With PLY-Bearing Liposomes Induces Phenotypic Changes in Macrophages

**Figure 3:**
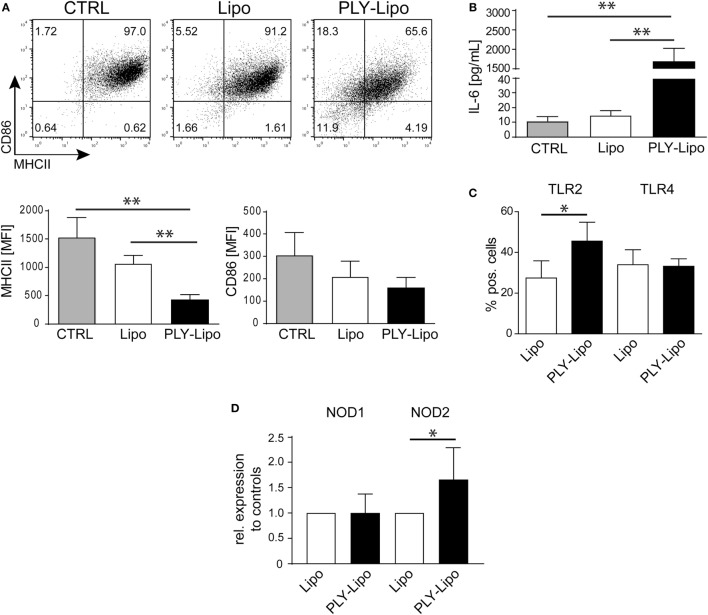
Pneumolysin (PLY)-bearing liposomes induce phenotypic changes in human macrophages *in vitro*. PLY-bearing liposomes (PLY-Lipo) were produced by pre-incubation of 40 µg/ml cholesterol:sphingomyelin liposomes with 4 µg/ml PLY for 10 min at room-temperature. This protocol efficiently sequesters the total amount of added toxin and neither cytotoxicity nor binding of free PLY to cultured cells is observed (see Figure 2A). **(A)** PLY-liposomes induce a downregulation of MHCII and CD86 in macrophages. Day 7 M-CSF differentiated human macrophages were stimulated with PLY-liposomes (PLY-Lipo), liposomes without PLY (Lipo) or left untreated (CTRL) and analyzed by FACS gated for CD14^+^ macrophages after 48 h. PLY-Lipo stimulated macrophages showed a marked downregulation of MHCII and CD86 as compared to control cells (untreated or liposomes only; Lipo). A representative FACS blot of one of three independent experiments is shown. Mean fluorescence intensity of MHCII and CD86 of CD14^+^ gated cells of three independent experiments are shown (mean ± SD, *n* = 5; *p < 0.05; unpaired *t*-test; two-tailed). **(B)** PLY-liposomes induce IL-6 production in macrophages. Human macrophages were stimulated as in **(A)** and IL-6 production was determined after 48 h from culture supernatants performing Bio-Plex assays (BioRad). IL-6 production is shown to be significantly enhanced in PLY-Lipo stimulated macrophages (mean ± SD; *n* = 7; **p < 0.005; unpaired *t*-test; two-tailed). **(C)** PLY-liposomes induce TLR2 expression in macrophages. Day 7 M-CSF differentiated human macrophages were stimulated with PLY-Lipo or liposomes only (Lipo) and analyzed by FACS gated for CD14^+^ macrophages after 48 h. PLY-Lipo stimulated macrophages showed a significant increase in TLR2 as compared to control cells (liposomes only; Lipo). Percentages of TLR2- and TLR4-expressing CD14^+^ macrophages are shown (mean ± SD; *n* = 6; *p < 0.05; paired *t*-test; two-tailed). **(D)** PLY-liposomes induced macrophages express increased levels of NOD2. Day 7 M-CSF differentiated human macrophages were stimulated with PLY-Lipo or liposomes only (Lipo) for 48 h. RNA was isolated and RT-PCR was performed to analyze expression levels of NOD1 and NOD2 in these cells. PLY-liposome induced macrophages showed a significant increase in NOD2 expression as compared to control cells (liposomes only; Lipo). NOD1 levels remained unchanged (mean ± SD; *n* = 7; *p < 0.05; paired *t*-test; two-tailed).

Next, we investigated whether PLY-liposomes induce changes in surface marker expression and cytokine production similar to PLY-microvesicle induced changes (Figure 1). These liposomes, which were shown to efficiently bind PLY (34), would serve as presenting platforms for detoxified PLY in analogy to membrane-repair induced microvesicles. PLY-liposome stimulation induced a downregulation of MHCII and CD86 (Figure 3A). As observed for PLY-microvesicle-stimulated macrophages, the M2 phenotype-associated expression of CD206 and CD163 was also not induced in PLY-liposome stimulated cells (Figure S1B in Supplementary Material). PLY-liposome stimulated macrophages showed a robust increase in IL-6 production, comparable to IL-6 induction in PLY-microvesicle simulated macrophages (Figure 3B versus Figure 1C). Thus, PLY-liposome stimulation of macrophages phenocopies the effects observed with host-derived PLY-microvesicles in terms of downregulation of MHCII and IL-6 induction.

The induction of IL-6 secretion argues against an anti-inflammatory M2-macrophage phenotype in which IL6 is not induced. However, IL-6 levels are also not as elevated as in a pro-inflammatory M1-macrophage (Figure S3A in Supplementary Material).

To exclude that contaminating active PLY induces the observed effects on macrophages, we made use of a non-toxic mutated version of PLY (A146R147; amino acid deletions A146, R147; ΔPLY). ΔPLY protein mutant is capable of forming oligomers on membranes but lacks pore-forming activity (7, 27). Thus, ΔPLY protein was incubated with liposomes in analogy to the protocol used for wild-type PLY. Next, macrophages were stimulated with ΔPLY-liposomes or ΔPLY protein alone. After 48 h, CD14^+^ gated macrophages were analyzed for MHCII and CD86 expression. ΔPLY-liposome stimulated macrophages showed a downregulation of MHCII and CD86 comparable to wild-type PLY liposome stimulated macrophages (Figure S3B in Supplementary Material versus Figure 3A). Thus, pore-forming activity is not accounting for phenotypic changes in macrophages stimulated with PLY. Moreover, ΔPLY protein alone stimulated macrophages exhibit a similar phenotype. High IL-6 secretion in ΔPLY-liposome or ΔPLY alone stimulated macrophages showed no differences to PLY-liposome stimulated macrophages (Figure S3C in Supplementary Material).

Pathogen-associated molecular patterns are sensed by macrophages through PRRs, e.g., TLRs, NLRs, and RLRs. Bacterial cell wall components, such as LPS from Gram-negative bacteria are recognized by TLR4, or lipoproteins from Gram-positive bacteria are recognized by TLR2. FACS analysis of PLY-liposome stimulated macrophages revealed a significant increase of TLR2 expression, whereas TLR4 levels remained constant (Figure 3C). RT-PCR analysis shows no increased TLR2 transcript abundance in PLY-liposome stimulated macrophages, indicating that elevated TLR2 surface levels are due to a posttranscriptional effect (Figure S4A in Supplementary Material). Moreover, also TLR1, TLR4, TLR5, and TLR6 expression levels remained unchanged in PLY-liposome stimulated macrophages as compared to control cells (Figure S4A in Supplementary Material). Detection of cell wall component PGN from Gram-positive bacteria is executed by NLRs, i.e., NOD1 and NOD2 (38). In particular, NOD2 was shown to sense MDP, whereas NOD1 detects muropeptides found mostly in PGN from Gram-negative bacterial species (9–11). RT-PCR analysis of PLY-liposome induced macrophages showed a significant increased expression of NOD2, but not NOD1, as compared to control macrophages (Figure 3D). These data suggest that inactivated PLY-pores on liposomes induce a specific CD14^+^MHCII^low^CD86^low^TLR2^+^NOD2^+^ macrophage phenotype distinct from M1 or M2 phenotypic polarization. These data also demonstrate that the signal is elicited by the inactivated toxin and not by constituents of the cell-derived, membrane repair-induced microvesicles.

### PLY-Liposome Pre-Stimulated Macrophages Show a Biased Response to Gram-Positive Versus Gram-Negative Bacterial Ligands

**Figure 4:**
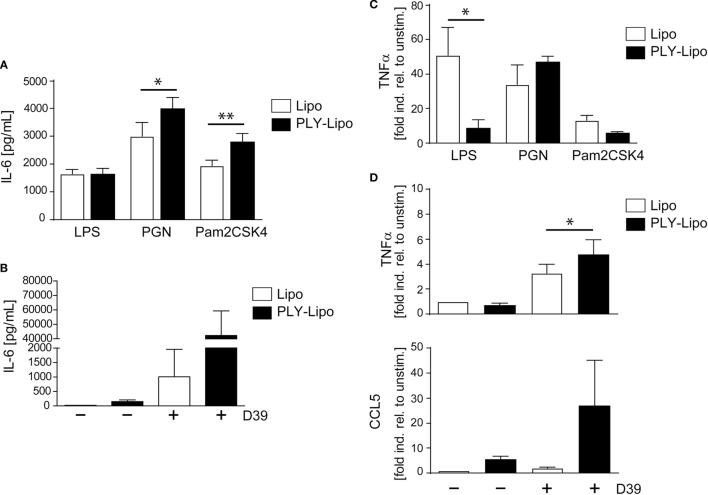
Pneumolysin (PLY)-liposome induced macrophages respond differentially to PRR ligands. Day 7 M-CSF induced human macrophages were stimulated with PLY-liposomes (PLY-Lipo) or liposomes only (Lipo) for 48 h. After stimulation, cell media was removed, macrophages were washed once, and re-stimulated with a TLR4 ligand (LPS), a TLR2 ligand (Pam2CSK4), a NLR ligand (PGN), or supernatant from *Streptococcus pneumonia* strain *D39* (*D39*). **(A,B)** PLY-liposome induced macrophages show an enhanced response to Gram-positive PRR/TLR ligands. Re-stimulation of PLY-Lipo induced macrophages was performed with 100 ng/ml LPS, 1 µg/ml Pam2CSK4, 1 µg/ml PGN, or supernatants from *Streptococcus pneumonia D39* for 6 h. IL-6 production was analyzed by collecting supernatants at 6 h and performing Bio-Plex measurements (mean ± SD, *n* = 6; *p < 0.05, **p < 0.005, paired *t*-test, two-tailed). **(C,D)** TNFα upregulation in PLY-liposome induced macrophages is increased in response to Gram-positive PRR ligands. **(C)** PLY-Lipo induced macrophages were re-stimulated as in **(A)** and RNA was isolated at time points 0 and 6 h. RT-PCR analysis was performed using primers for TNFα and SYBR green RT-PCR kit. Glyceraldehyde 3-phosphate dehydrogenase (GAPDH) served as a reference gene (mean ± SD, five donors; *p < 0.05, paired *t*-test, one-tailed). **(D)** PLY-Lipo induced macrophages were re-stimulated with supernatants from *S. pneumoniae* strain *D39* and RNA was isolated at time points 0 and 6 h. RT-PCR analysis was performed using primers for TNFα and CCL5. Glyceraldehyde 3-phosphate dehydrogenase (GAPDH) served as a reference gene. Mean ± SD of five independent experiments and donors are shown (*p < 0.05, paired *t*-test, one-tailed).

Macrophages, which recognize pathogenic bacteria by PRRs respond with phagocytosis and/or inflammatory cytokine production. Macrophage polarization induces an increase (M1) or decrease (M2) in phagocytosis (39–41). Analysis of phagocytic capacity in PLY-liposome induced macrophages using GFP-*E. coli* showed an equally high phagocytotic efficiency in PLY-liposome treated versus empty liposome treated macrophages (Figure S4B in Supplementary Material). Since PLY-liposome stimulation of macrophages induces TLR2 in favor of TLR4, we examined how PLY-liposome induced macrophages respond to TLR ligation. Re-stimulation of PLY-liposome induced macrophages with LPS led to identical IL-6 secretion as in cells stimulated with empty liposomes (Figure 4A). In contrast, re-stimulation of PLY-liposome induced macrophages with Pam2CSK4, a synthetic diacylated lipopeptide that is sensed by TLR2, induced a significantly enhanced IL-6 production as compared to control macrophages (Figure 4A). Moreover, re-stimulation of these macrophages with PGN led to a significantly enhanced IL-6 production (Figure 4A). Stimulation of PLY-liposome induced macrophages with supernatants from *S. pneumoniae D39* (virulent serotype 2) induced a massive IL-6 response, higher than in cells treated with empty liposomes (Figure 4B). TNFα represents another important cytokine induced in pneumococcal pneumonia (42). LPS re-stimulation of empty liposome-induced macrophages showed immediate TNFα upregulation as expected; however, no induction of TNFα in PLY-liposome induced macrophages was observed (Figure 4C). When re-stimulated with PGN, the induction of TNFα within 6 h was increased in PLY-liposome induced macrophages versus control cells, although not significantly (Figure 4C). In contrast, re-stimulation of these cells with Pam2CSK4 had no significant effect on the expression levels of TNFα (Figure 4C). Re-stimulation with *S. pneumoniae D39* supernatants confirmed the observed preferential reaction to Gram-positive bacterial ligands of PLY-liposome induced macrophages and showed an enhanced induction of TNFα (Figure 4D). In addition, the expression of CCL5, an important chemokine, which attracts neutrophils in pneumococcal infections (43), was strongly upregulated in re-stimulated PLY-liposome-induced macrophages, whereas controls did not induce CCL5 expression (Figure 4D). In contrast, when challenged with LPS, TNFα induction was inhibited and no increase in IL-6 was observed. These data suggest that PLY-liposome induced CD14^+^MHCII^low^CD86^low^TLR2^+^NOD2^+^ macrophages display an enhanced response to Gram-positive ligands and a blunted response to Gram-negative microbial products. Our data thus indicate that macrophages can be polarized in response to bacterial toxins, even when they are presented in an inactive state.

Toll-like receptors signaling provides positive signals in immune cell activation and is counter-regulated by a negative feed-back loop executed by Suppressor of Cytokine Signaling (SOCS) and Signal Transducer and Activator of Transcription (STAT) proteins (44). Therefore, we analyzed these regulatory molecules in CD14^+^MHCII^low^CD86^low^TLR2^+^ macrophages. Compared with control cells, PLY-liposome induced macrophages display increased steady-state levels of SOCS1, SOCS3, and STAT1, which may explain the blunted response to LPS by these cells (Figure S4C in Supplementary Material).

### Stimulation of Macrophages With Diverse Pore-Forming Toxin-Bearing Liposomes Induces Selective Phenotypic Changes

**Figure 5:**
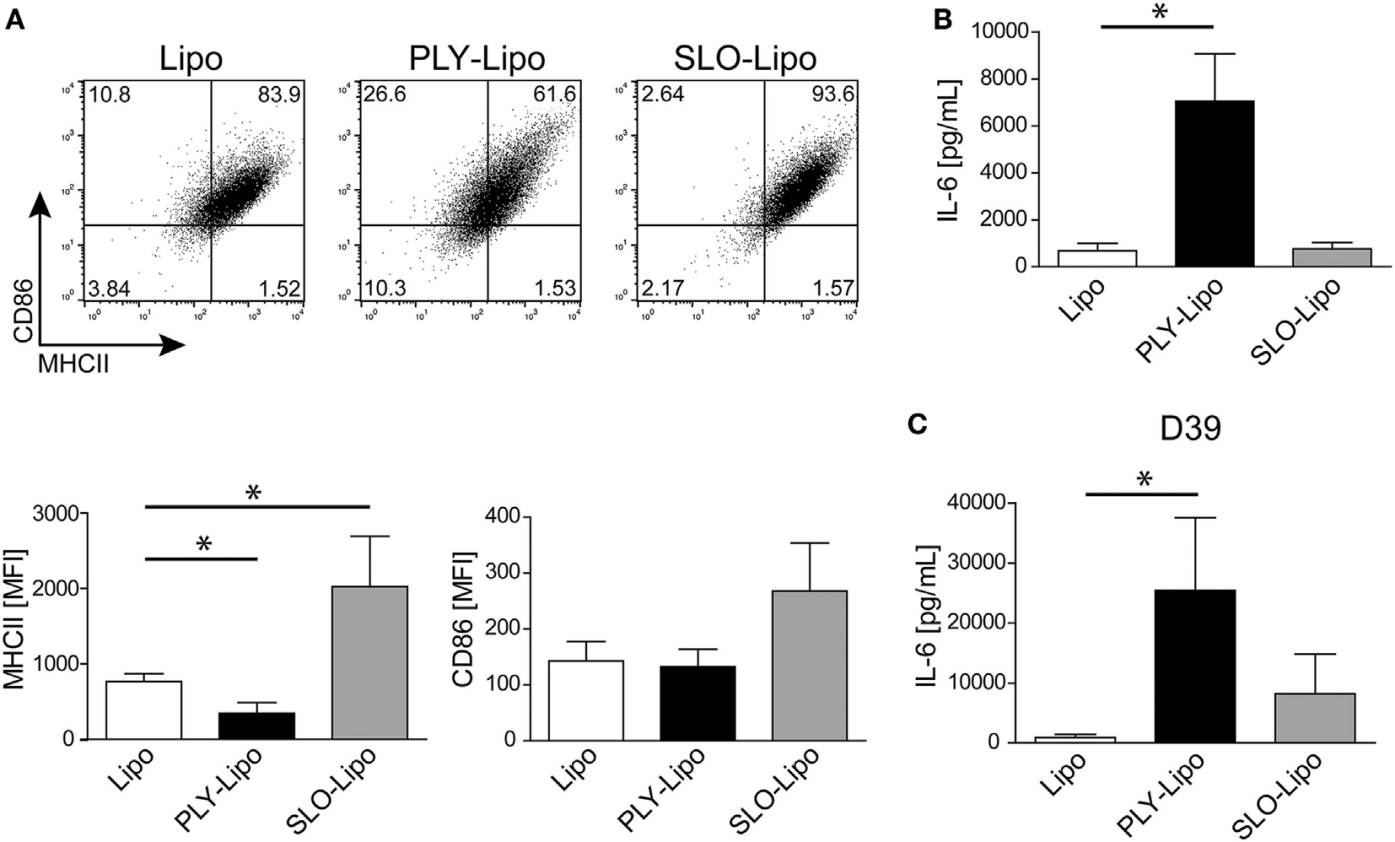
Streptolysin O (SLO)-bearing liposomes-induced macrophages display a phenotype distinct from Pneumolysin (PLY)-liposome induced macrophages. Day 7 M-CSF induced human macrophages were stimulated with PLY-liposomes (PLY-Lipo) or SLO-Liposomes (SLO-Lipo) for 48 h. Equal amounts (4 µg/ml) of PLY or SLO protein were incubated with 40 µg/ml liposomes for 10 min at room-temperature before addition to the cells. **(A)** SLO-liposomes induce an upregulation of MHCII and CD86 in macrophages. Macrophages were stimulated with PLY-Lipo, SLO-Lipo, or liposomes without pore-forming toxin (Lipo) and analyzed by FACS gated for CD14^+^ cells after 48 h. Expression levels of MHCII and CD86 were evaluated compared to control cells (liposomes only; Lipo). A representative FACS blot of one of three independent experiments is shown. Mean fluorescence intensity of MHCII and CD86 of CD14^+^ gated cells of three independent experiments are shown (mean ± SD, *n* = 6; *p < 0.05, unpaired *t*-test, two-tailed). **(B)** SLO-liposomes stimulation does not induce IL-6 production in macrophages. Human macrophages were stimulated as in **(A)** and IL-6 production was determined after 48 h from culture supernatants performing Bio-Plex assay (mean ± SD, *n* = 5; *p < 0.05, paired *t*-test, two-tailed). **(C)** Re-stimulation of SLO-Lipo induced macrophages with *Streptococcus pneumonia* supernatants. Human macrophages were stimulated with PLY-liposomes (PLY-Lipo) or SLO-liposomes (SLO-Lipo) for 48 h. After stimulation, cell media was removed, macrophages were washed once, and re-stimulated with supernatant from *S. pneumonia* strain *D39* for 6 h. IL-6 production was analyzed by performing Bio-Plex measurements (mean ± SD, *n* = 4; *p < 0.05, paired *t*-test, two-tailed).

Since numerous Gram-positive bacteria produce specific CDCs, we analyzed the macrophages’ response to SLO (from *S. pyogenes*). In analogy to PLY, SLO-pores are efficiently sequestered by liposomes (34). Thus, we generated SLO-bearing liposomes and stimulated macrophages according to our previous protocols. In contrast to PLY-liposomes, SLO-liposomes induced an upregulation of MHCII and CD86 and no increase in IL-6 secretion (Figures 5A,B). Thus, the macrophage phenotype after stimulation with SLO-liposomes (CD14^+^MCHII^hi^CD86^hi^) clearly differed from stimulation with PLY-liposomes (CD14^+^MCHII^low^CD86^low^, Figure 3A). Cytokine profiling of SLO-liposome stimulated macrophages revealed a distinct cytokine production pattern as compared to PLY-liposome stimulated macrophages (Figure S5A in Supplementary Material). SLO-liposome stimulated macrophages show a moderate upregulation of IL-10, CCL8, and CCL15 in comparison to control cells. In line with a different macrophage polarization, re-stimulation of SLO-liposome induced macrophages with *S. pneumoniae D39* supernatants resulted in a lower secretion of IL-6 than in those stimulated with PLY-liposomes (Figure 5C). Interestingly, IL-6 secretion of SLO-liposome stimulated macrophages after re-stimulation exceeded that of control cells (9.2-fold increase versus control, Figure 5C). Induction of TNFα in SLO-induced macrophages after re-stimulation was comparable to PLY-liposome induced macrophages, whereas there was no induction of CCL5 in SLO-induced macrophages (Figure S5B in Supplementary Material). Interestingly, SLO-induced macrophages produced high amounts of IL-6 when re-stimulated with LPS. Taken together, these data show that SLO-liposomes induce macrophage polarization to a CD14^+^MHCII^hi^CD86^hi^ phenotype distinct from PLY-liposome induced macrophages (CD14^+^MHCII^low^CD86^low^). Moreover, SLO-liposome induced macrophages show an enhanced IL-6 secretion in response to LPS, but only a moderate IL-6 production when re-challenged with *S. pneumoniae D39* supernatants (Figure S5C in Supplementary Material; Figure 5C). Thus, these data indicate that neutralized PFTs convey distinct polarization signals.

## Discussion

We show that inactive PFTs, sequestered by liposomes or shed on microvesicles during membrane repair processes, polarize macrophages to enhance responses to Gram-positive PAMPs.

**Figure 6:**
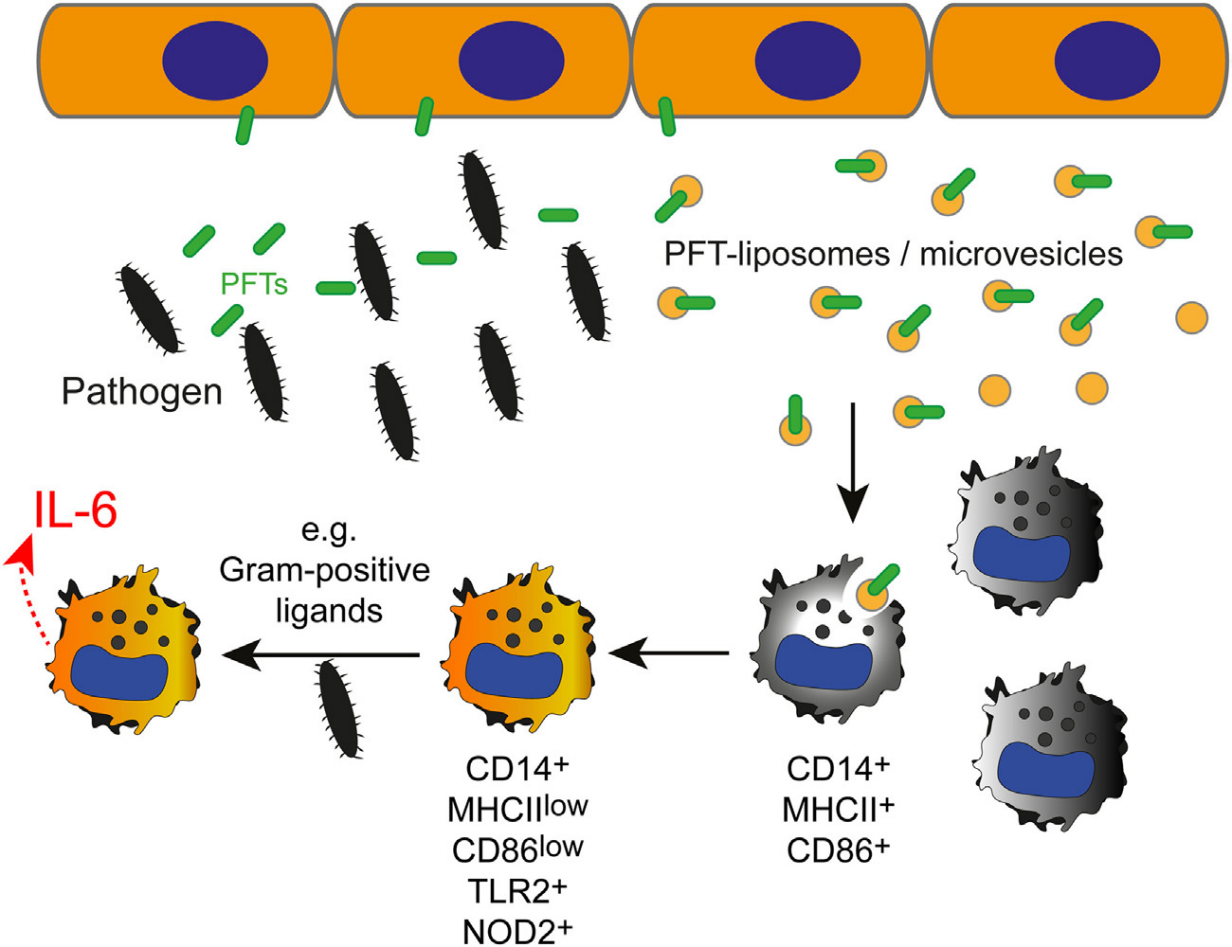
The effect of inactivated pneumolysin (PLY)-pores on macrophages in an inflammatory environment. Pore-forming toxins (PFTs), such as PLY perforate the plasmalemma of host cells. Membrane repair is initiated and toxin pores are shed into the extracellular space in the form of microvesicles (7). Alternatively, the administration of specific liposomes sequesters the pore-forming toxin and protects the host cell from injury (34). PLY-pores on liposomes or on microvesicles (PFT-liposomes/microvesicles) are sensed by macrophages and prime macrophages to preferentially react against pore-forming toxin-producing Gram-positive bacteria, i.e., *Streptococcus pneumoniae*, with an increased production of IL-6 in response to PRR ligation (i.e., TLR2, NOD2) by Gram-positive ligands.

Although the innate immune response to different bacteria is thought to be non-specific, this short-cut in toxin identification appears to enable an early adaption of the immune response to the pathogen (see model in Figure 6).

The type of polarization of macrophages is critical for the resolution or non-resolution of an inflammatory condition. Polarization into distinct subpopulations depends on the inflammatory environment (i.e., cytokines, microbial products, immunocomplexes) (23). We show that PLY-liposome stimulated macrophages polarize to a CD14^+^MHCII^low^CD86^low^TLR2^hi^NOD2^+^ phenotype. M1-macrophages express high levels of MHCII and CD86 whereas downregulation of these molecules was shown to be associated with M2-polarization (45, 46). It is important to note that none of the other established markers for M2 macrophage polarization (i.e., CD206, CD163) are upregulated after stimulation with sequestered PLY. The cytokine pattern elicited by sequestered PLY resembles a pro-inflammatory macrophage signature, i.e., production of IL-1β, TNFα, IL-6, CCL5, CCL8, and CCL1, for attraction of innate and adaptive immune cells. Thus, the macrophage phenotype induced by inactive PLY is novel, but closely resembles a pro-inflammatory phenotype (despite the downregulation of MHCII and CD86) at least in response to Gram-positive PAMPs.

The pore-forming activity of PLY is not essential for initiating the phenotypic changes in macrophages, as a non-hemolytic ΔPLY mutant protein, similarly induces downregulation of MHCII and CD86 and secretion of IL-6. These data supports that PLY protein itself, either neutralized on liposomes or shed microvesicles, can be recognized by macrophages and induce macrophage polarization. These data are in line with the immunogenic properties of PLY protein, which have been proposed for use in pneumococcal vaccines (27, 47).

Whereas TLR4 levels remain unchanged, TLR2 and NOD2 expression is increased in sequestered PLY stimulated macrophages. The importance of TLR2 in the defense against Gram-positive bacteria is well documented: TLR2-deficient mice are more susceptible to *S. pneumoniae* infection (48). Re-stimulation of these macrophages with Gram-positive PAMPs (*S. pneumoniae D39* supernatants) induced enhanced production of IL-6, TNFα, and CCL5. These cytokines have been assigned important functions in the resolution of *S. pneumoniae* infections (43, 49, 50). Although TLR4 expression does not change in CD14^+^MHCII^low^CD86^low^ macrophages, these cells show a blunted TNFα induction in response to Gram-negative LPS, whereas IL-6 secretion in response to LPS is not altered.

CD14^+^MHCII^low^CD86^low^TLR2^hi^NOD2^+^ macrophages display an increased expression of SOCS1, SOCS3, and STAT1, known regulators of macrophage functions/cytokine responses. SOCS1 was shown to inhibit TLR4 signaling, whereas SOCS3 regulates pro-inflammatory functions (51–53). Thus, an elevated expression of SOCS1 in PLY-liposome induced macrophages may reduce the LPS response of these cells with regard to TNFα induction.

It has been suggested that the interaction of PLY with TLR4 is critically involved in the innate immune response to *S. pneumoniae*: TLR4-mutant mice were more susceptible to lethal infection with PLY-positive pneumococci (54). LPS tolerance is induced *via* TLR4 signaling and is known to reduce phagocytic activity of macrophages and lower IL-6 secretion after LPS re-stimulation (55, 56). PLY-liposome induced macrophages possessed identical phagocytic capacity as control cells. Moreover, no differences in IL-6 response to LPS re-stimulation were observed. Thus, it is unlikely that the CD14^+^MHCII^low^CD86^low^TLR2^hi^ phenotype results from excessive PLY-TLR4 signaling, since this would lead to LPS tolerance and no IL-6 response after re-stimulation with LPS.

Host cell membrane repair mechanisms release microvesicles that bear inactive PFT-pores (5). PLY-microvesicles induce macrophage polarization to a CD14^+^MHCII^low^CD86^low^ phenotype, which leads to a preferential response to Gram-positive PAMPs. Thus, inactive PLY-pores on microvesicles from membrane repair events serve as DAMPs, which might alert immune cells and shape the type of immune response elicited by infection with *S. pneumoniae*. If PFT-microvesicles are present in sufficient amounts *in vivo* to polarize macrophages in bacterial infections remains to be determined. However, in the local infection microenvironment, e.g., in proximity to epithelial barriers, macrophages would be sensing sufficient amounts of PLY-microvesicles to induce polarization, as described for endogenous molecules released by damaged cells (DAMPs) that signal to macrophages (57).

Streptolysin O, produced by *S. pyogenes*, is another prominent pore-forming toxin of the cholesterol-dependent cytolysin-family with similarities to PLY (1). Contrary to PLY-liposome polarization of macrophages, stimulation with SLO–liposomes induces a CD14^+^MHCII^hi^CD86^hi^ phenotype, low levels of IL-6, and an enhanced response against Gram-negative LPS. Moreover, the IL-6 production of CD14^+^MHCII^hi^CD86^hi^ macrophages in response to *S. pneumoniae* supernatant stimulation was similar to control macrophages. Thus, the macrophage polarization in response to inactive PLY on liposomes or microvesicles is unique and clearly differs from inactive SLO. These data suggests different recognition receptors on macrophages for distinct PFTs, which remain to be identified. Future experiments will also elucidate the protein domains/structures of distinct PFTs that drive macrophage polarization.

Our work shows that PLY-microvesicles or PLY-liposomes act as alarm (DAMPs)-like signals that polarize macrophages to preferentially respond to Gram-positive PAMPs. The liposomes used in this study, which sequester and neutralize PFTs, i.e., PLY or SLO, are currently in a phase II clinical trial as adjunctive therapy to antibiotics in severe streptococcal pneumonia (34). Our data provide a novel mode-of-action of these liposomes with trapped PFTs, especially on immune cells, which are key players in the host defense and in the coordination of immune responses against bacterial infections. Importantly, such inactivated PLY-pores on microvesicles as well as on liposomes lack cell damaging capacity and thus are sensed without danger by immune cells. The fact that PLY-liposomes phenocopy the activation of macrophages with PLY-microvesicles suggests that a key signal for macrophage activation is delivered by the inactivated PLY-pores alone and not by microvesicle-associated proteins.

Thus, macrophages in immune responses to streptococcal infection may be activated/polarized through this novel signaling mechanism in order to initiate specific immune responses, presumably for the efficient clearing of distinct bacterial pathogens.

## Author Contributions

RK designed research, performed most experiments, analyzed the data, supervised the project, and wrote the manuscript; YL, HB, HW, RS, VB, and PD performed experiments; TP contributed to reagents and methodology; TM contributed to reagents and reviewed the manuscript; EB and AD analyzed data and contributed in writing the manuscript. All authors discussed and approved the results presented in the manuscript.

## Conflict of Interest Statement

The authors declare that the research was conducted in the absence of any commercial or financial relationships that could be construed as a potential conflict of interest.
