# Supplementary material for: Host-Derived Microvesicles Carrying Bacterial Pore-Forming Toxins Deliver Signals to Macrophages: A Novel Mechanism of Shaping Immune Responses
Source: Front Immunol. 2018 Jul 27;9:1688. doi: 10.3389/fimmu.2018.01688 (PMC6072879; doi:10.3389/fimmu.2018.01688)
Supplement: Supplementary file 1 [file presentation_1.PDF]

Supplementary Material

Host-derived microvesicles carrying bacterial pore-forming toxins deliver signals to macrophages: a novel mechanism of shaping immune responses

René Köffel [1*], Heidi Wolfmeier [1†] , Yu Larpin [1], Hervé Besançon [1], Roman Schoenauer [1], Viktoria S. Babiychuk [1], Patrick Drücker [1], Thomas Pabst [2], Timothy J. Mitchell [3], Eduard B. Babiychuk [1], Annette Draeger [1]

[1] Institute of Anatomy, University of Bern, Bern, Switzerland.

[2] Department of Medical Oncology, University Hospital Bern, Bern, Switzerland.

[3] Institute of Microbiology and Infection, College of Medical and Dental Sciences, University of Birmingham, Edgbaston, Birmingham, United Kingdom.

[†]Current address: Department of Microbiology and Immunology, University of British Columbia, Vancouver, Canada

*** Correspondence:**

René Köffel

rene.koeffel@ana.unibe.ch (RK)

Short title: Neutralized pore-forming toxins shape macrophage phenotypes

Supplementary Figures: 5

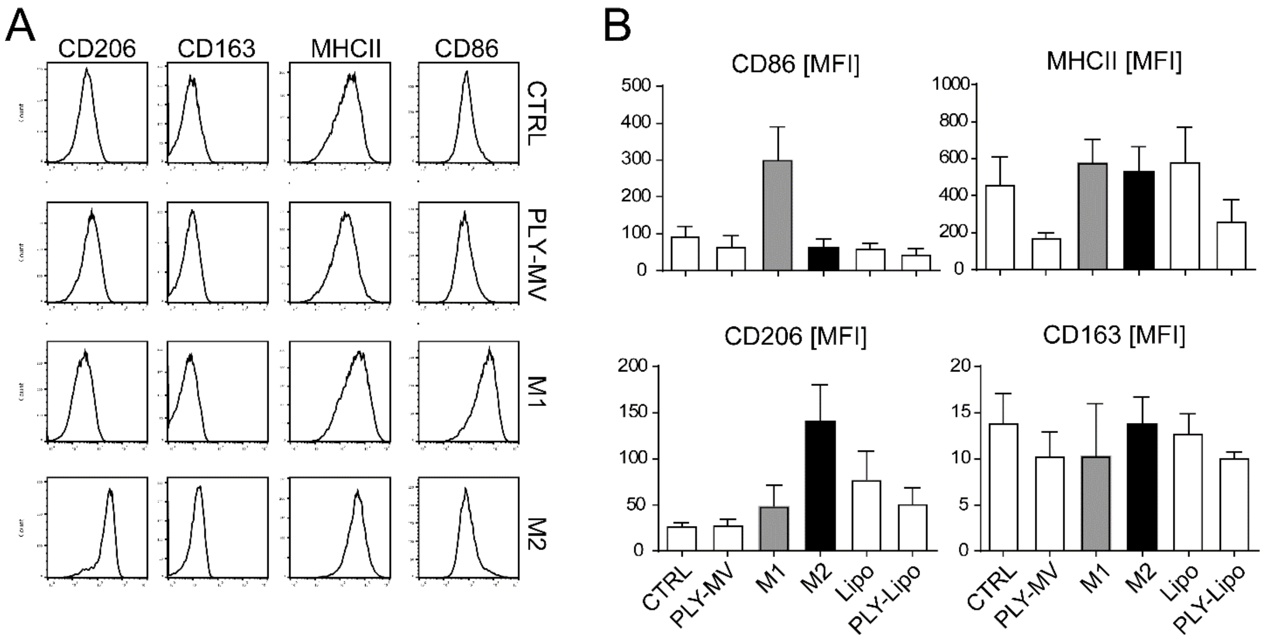

Supplementary Figure 1. Analysis of M1- and M2-marker expression in pneumolysin-microvesicle or pneumolysin-liposome stimulated macrophages. (A) Pneumolysin-microvesicle (PLY-MV) and control-treated (CTRL) macrophages were analyzed for the expression levels of M1 and M2 macrophage polarization markers on CD14+ gated populations. One representative blot of 5 independent experiments is shown. (B) Summary of macrophage marker expression of pneumolysin-liposome (PLY-Lipo), pneumolysin-microvesicle (PLY-MV), untreated (CTRL) or liposome alone (Lipo) stimulate macrophages compared with M1 and M2 polarized macrophages (Mean ± SD; n=5).

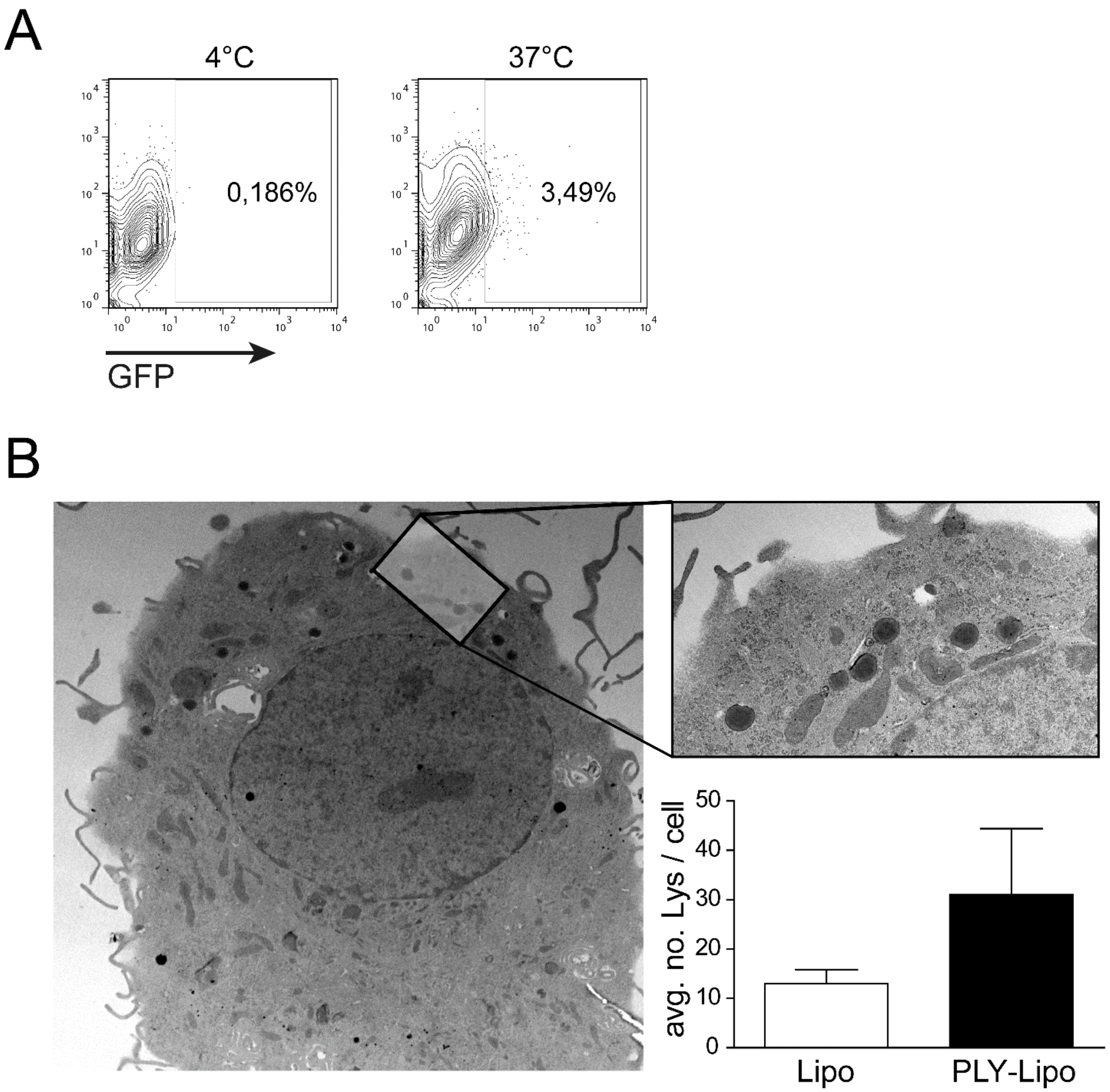

Supplementary Figure 2. Pneumolysin-liposomes induce lysosomes in macrophages. Day 7 M-CSF induced human macrophages were stimulated with PLY-liposomes (PLY-Lipo) or with liposomes alone (Lipo). (A) Uptake of EGFP-PLY-liposomes was analyzed after 4 hours stimulation by FACS (B) Lysosome numbers were analyzed after 4 hours stimulation. Cell media was removed, macrophages were washed once, and fixed with 2.5% Glutaraldehyde. Sample preparation for electron microscopy was performed according to standard protocols. Individual cells (n=10 / group) were analyzed for the presence of lysosomes. Mean ± SD of 2 independent experiments and donors are shown.

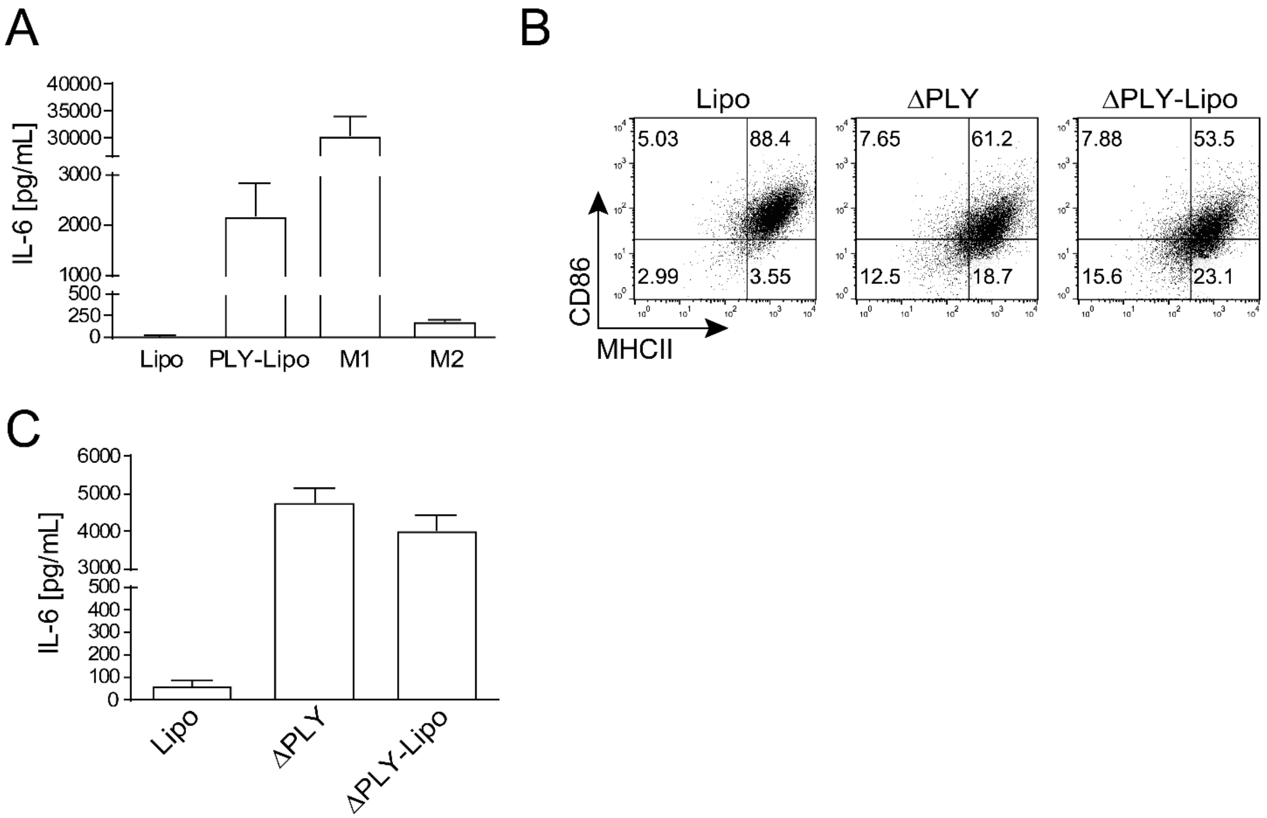

Supplementary Figure 3. Phenotypic characterization of pneumolysin-liposome induced macrophages. (A) IL-6 production of PLY-liposome stimulated, M1, or M2 polarized macrophages was determined after 48 hours from culture supernatants performing Bio-Plex assays (Mean ± SD; n=4) (B) **Non-hemolytic ΔA146R147 PLY protein induced macrophages.** Non-hemolytic ΔPLY protein mutant (ΔA146R147; amino acid deletions A146, R147) was shown to prominently bind to the plasma membrane but does not trigger plasmalemmal perforations (1,2). Day 7 M-CSF induced human macrophages were stimulated with ΔPLY protein alone (ΔPLY), ΔPLY protein bound to liposomes (ΔPLY-Lipo), or with liposomes alone (Lipo) for 48 hours. After stimulation, $CD14^+$ gated macrophages were analyzed for expression of CD86 and MHCII by FACS. A representative FACS blot of one of three independent experiments is shown. (C) **IL-6 production of non-hemolytic PLY protein mutant induced macrophages.** Human macrophages were stimulated with equal amounts of ΔPLY protein alone (ΔPLY), ΔPLY protein bound to liposomes (ΔPLY-Lipo), or with liposomes alone (Lipo) and IL-6 production was determined after 48 hours from culture supernatants performing Bio-Plex assays (Mean ± SD; n=3).

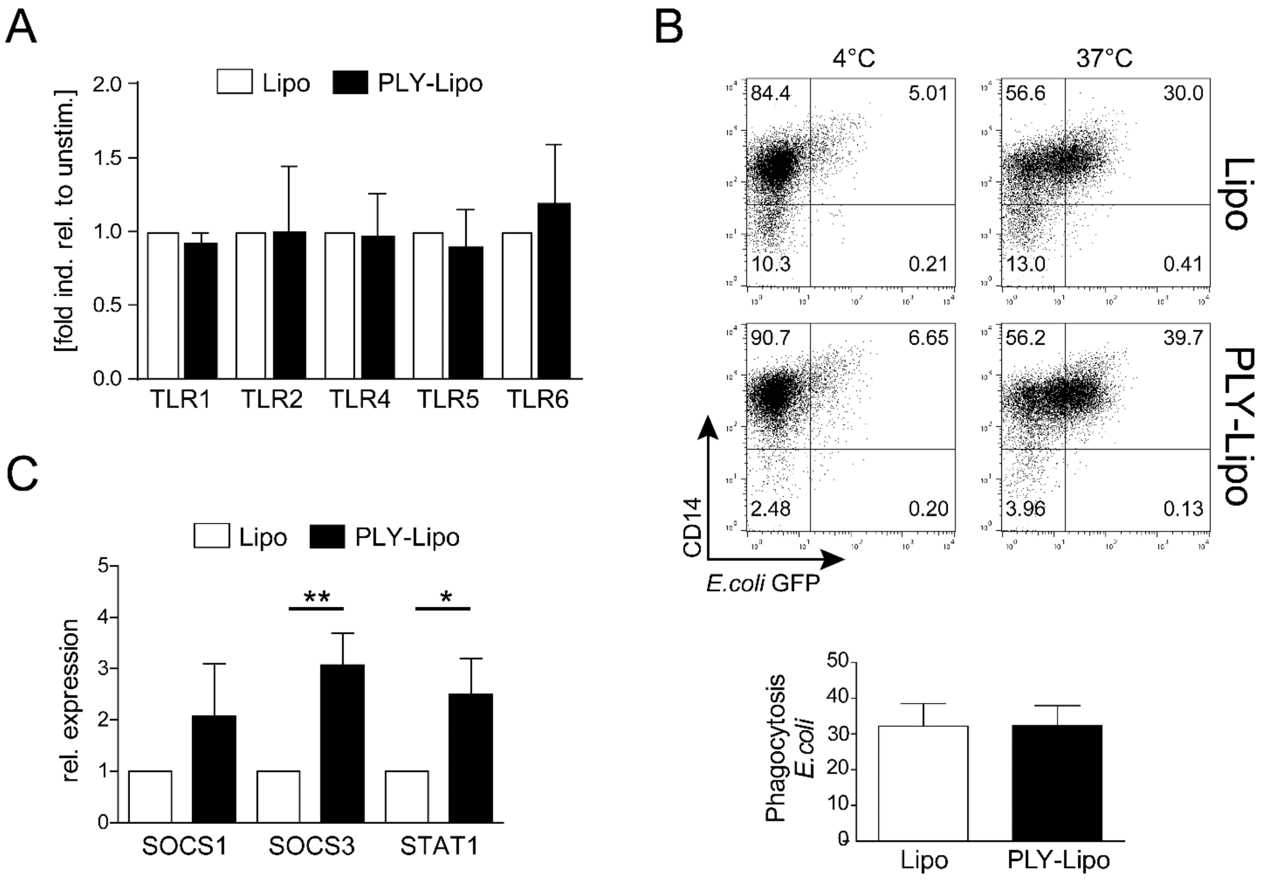

Supplementary Figure 4. Phenotypic characterization of pneumolysin-liposome induced macrophages. (A) TLR expression levels of PLY-liposome (PLY-Lipo) or liposome only (Lipo) stimulated macrophages was determined after 48 hours by RT-PCR (Mean ± SD; n=6) (B) **Phagocytic capacity of pneumolysin-liposome induced macrophages.** Day 7 M-CSF induced human macrophages were stimulated with PLY-Lipo or with liposomes only (Lipo) for 48 hours. After stimulation, the culture media was removed, macrophages washed once, and removed by treatment with 2mM EDTA/PBS for 10min. The phagocytosis assay was performed as described in (3). In brief, macrophages were analyzed for the uptake of *E.coli*-EGFP after 1.5 hours by FACS. Control cells were maintained on ice. A representative FACS blot of one of three independent experiments is shown. Mean ± SD of 3 independent experiments and donors are shown. (C) **RT-PCR of SOCS1, SOCS3, and STAT1 in pneumolysin-liposome induced macrophages.** SOCS and STAT are significantly upregulated after 48 hours of PLY-Lipo stimulation as compared to controls (liposome only; Lipo). (Mean ± SD; n=5; * p<0.05; ** p<0.01, paired t-test, one-tailed).

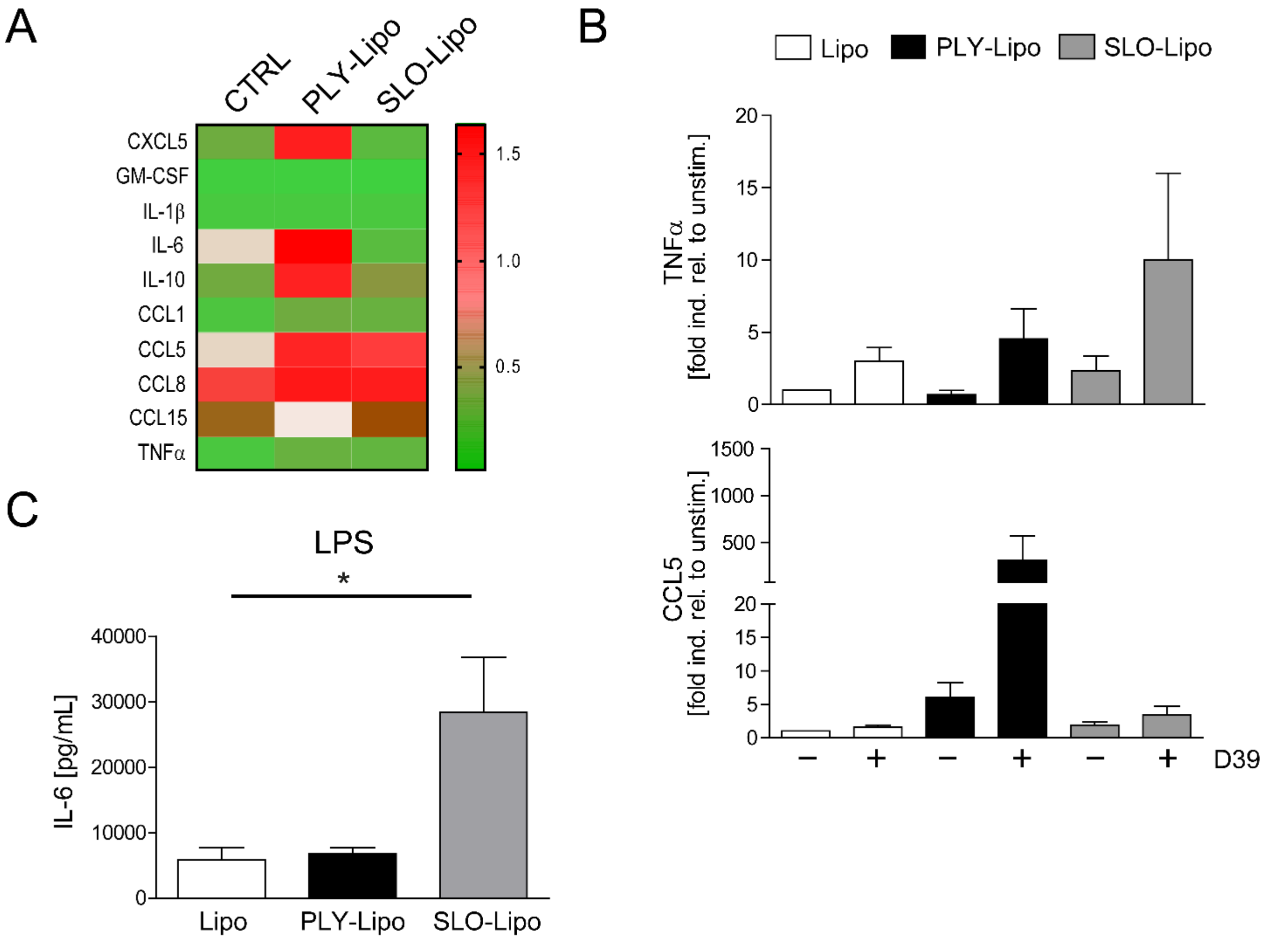

Supplementary Figure 5. (A) **Cytokine profiling of pneumolysin-liposome (PLY-Lipo) and streptolysin O-liposome (SLO-Lipo) stimulated macrophages versus control macrophages (untreated, CTRL).** Pooled supernatants from 2 independent experiments were analysed by human cytokine array for the detection of 42 human cytokines (Abcam). Densitometric analyses were accomplished using the ImageJ protein analyser macro (written by G. Carpentier, 2010; http://rsb.info.nih.gov/ij/macros/toolsets/Protein%20Array%20Analyzer.txt)). Values (arbitary units normalized to positive array controls) are displayed by as a heat map generated with the GraphPad Prism7 program. (B) **TNFα induction after re-stimulation with *Streptococcus pneumoniae D39*.** PLY-Lipo and SLO-Lipo induced macrophages were re-stimulated with supernatants from *Streptococcus pneumonia* D39 and RNA was isolated at time points 0 hours and 6 hours. RT-PCR analysis was performed using primers for TNFα and CCL5 using a SYBR green RT-PCR kit. Glyceraldehyde 3-phosphate dehydrogenase (GAPDH) served as a reference gene. Mean ± SD of 4 independent experiments are shown. (C) **IL-6 production after LPS re-stimulation of PLY-Lipo versus SLO-Lipo induced macrophages.** Human macrophages were stimulated with equal amounts of PLY-Lipo, SLO-Lipo, or liposomes only (Lipo) and IL-6 production was determined after 48 hours from culture supernatants performing Bio-Plex assays (Mean ± SD; n=5; *p<0.05, paired t-test, two-tailed).

Supplementary references

1. Kirkham L-AS, Kerr AR, Douce GR, Paterson GK, Dilts DA, Liu D-F, Mitchell TJ. Construction and immunological characterization of a novel nontoxic protective pneumolysin mutant for use in future pneumococcal vaccines. *Infect Immun* (2006) **74**:586–93. doi:10.1128/IAI.74.1.586-593.2006

2. Wolfmeier H, Radecke J, Schoenauer R, Koeffel R, Babiychuk VS, Drücker P, Hathaway LJ, Mitchell TJ, Zuber B, Draeger A, et al. Active release of pneumolysin prepores and pores by mammalian cells undergoing a Streptococcus pneumoniae attack. *Biochim Biophys Acta - Gen Subj* (2016) **1860**:2498–2509. doi:10.1016/j.bbagen.2016.07.022

3. Köffel R, Meshcheryakova A, Warszawska J, Hennig A, Wagner K, Jörgl A, Gubi D, Moser D, Hladik A, Hoffmann U, et al. Monocytic cell differentiation from band-stage neutrophils under inflammatory conditions via MKK6 activation. *Blood* (2014) **124**:2713–24. doi:10.1182/blood-2014-07-588178
